# Supplementary material for: Increase in Epithelial Permeability and Cell Metabolism by High Mobility Group Box 1, Inflammatory Cytokines and TPEN in Caco-2 Cells as a Novel Model of Inflammatory Bowel Disease
Source: Int J Mol Sci. 2020 Nov 10;21(22):8434. doi: 10.3390/ijms21228434 (PMC7696423; doi:10.3390/ijms21228434)
Supplement: Supplementary file 1 [file ijms-21-08434-s001.pdf]

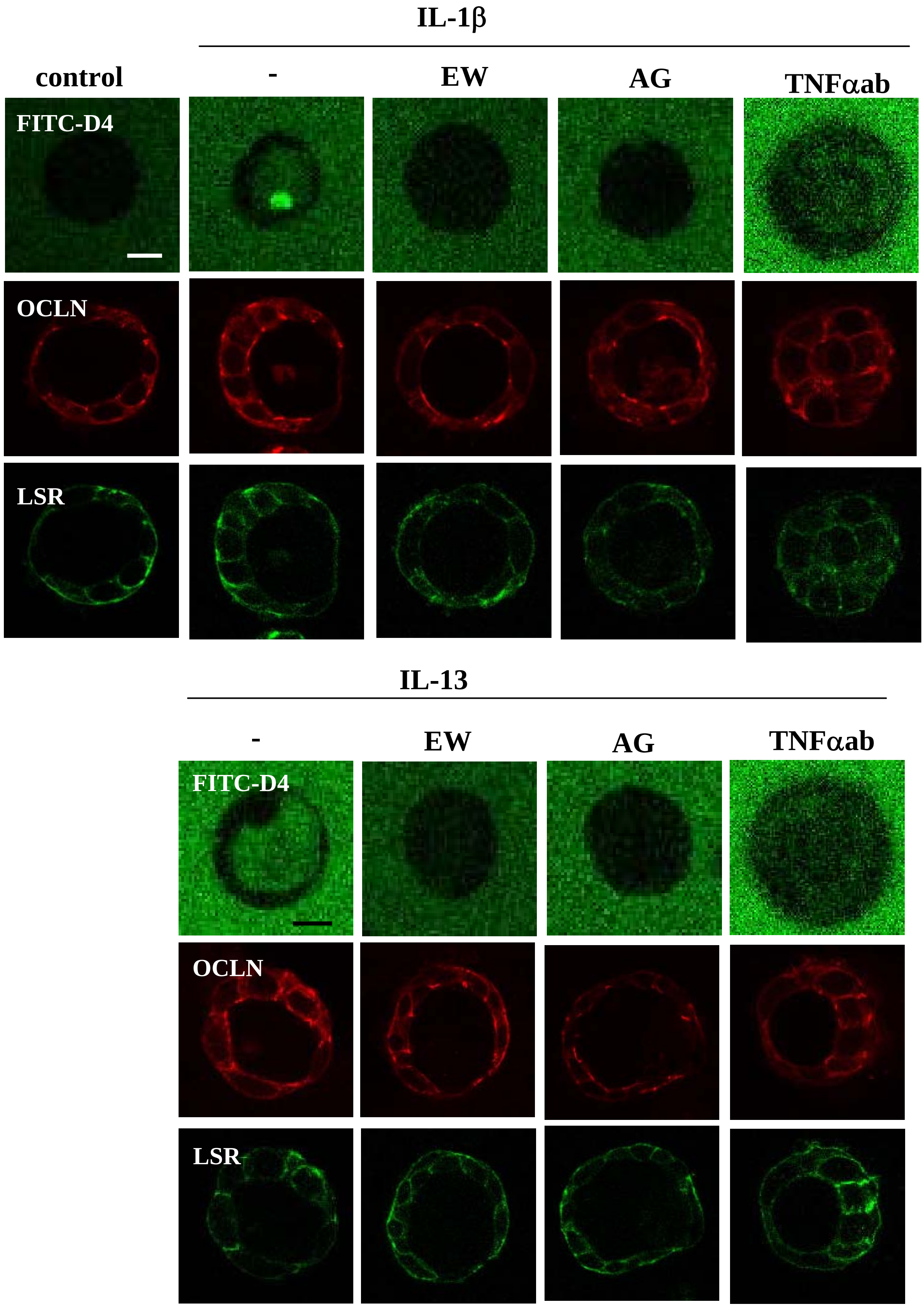

Supplemental Figure 1: Effects on tight junction molecules treated with IL-1β or IL-13 in 2.5D Matrigel culture of Caco-2 cells. Immunocytochemistry for OCLN, LSR and TRLC in 2.5D Matrigel culture of Caco-2 cells pretreated with 10 μM EW-7197, 10 μM AG-1478 or 40 μg/mL TNFα ab before treatment with 100 μg/mL IL-1β or 100 μg/mL IL-13. Scale bar: 20 μm.

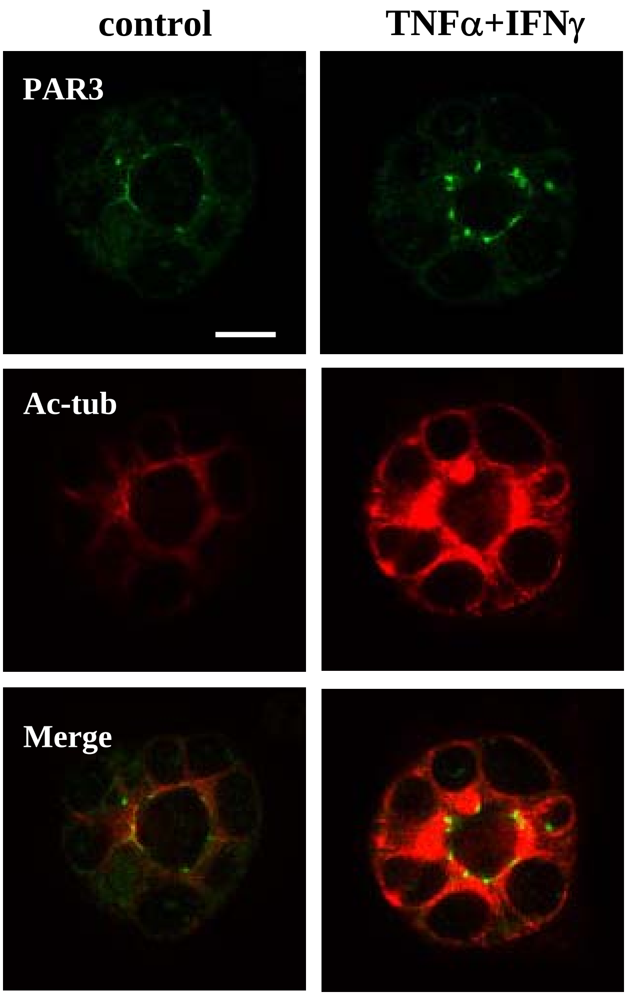

Supplemental Figure 2: Effects of treatments with TNFα and IFNγ on cilia formation in 2.5D Matrigel culture of Caco-2 cells. Immunocytochemistry for PAR3 and Ac-tub in 2.5D Matrigel culture of Caco-2 treated with 100 μg/mL TNFα and 100 μg/mL IFNγ. Scale bar: 20 μm.

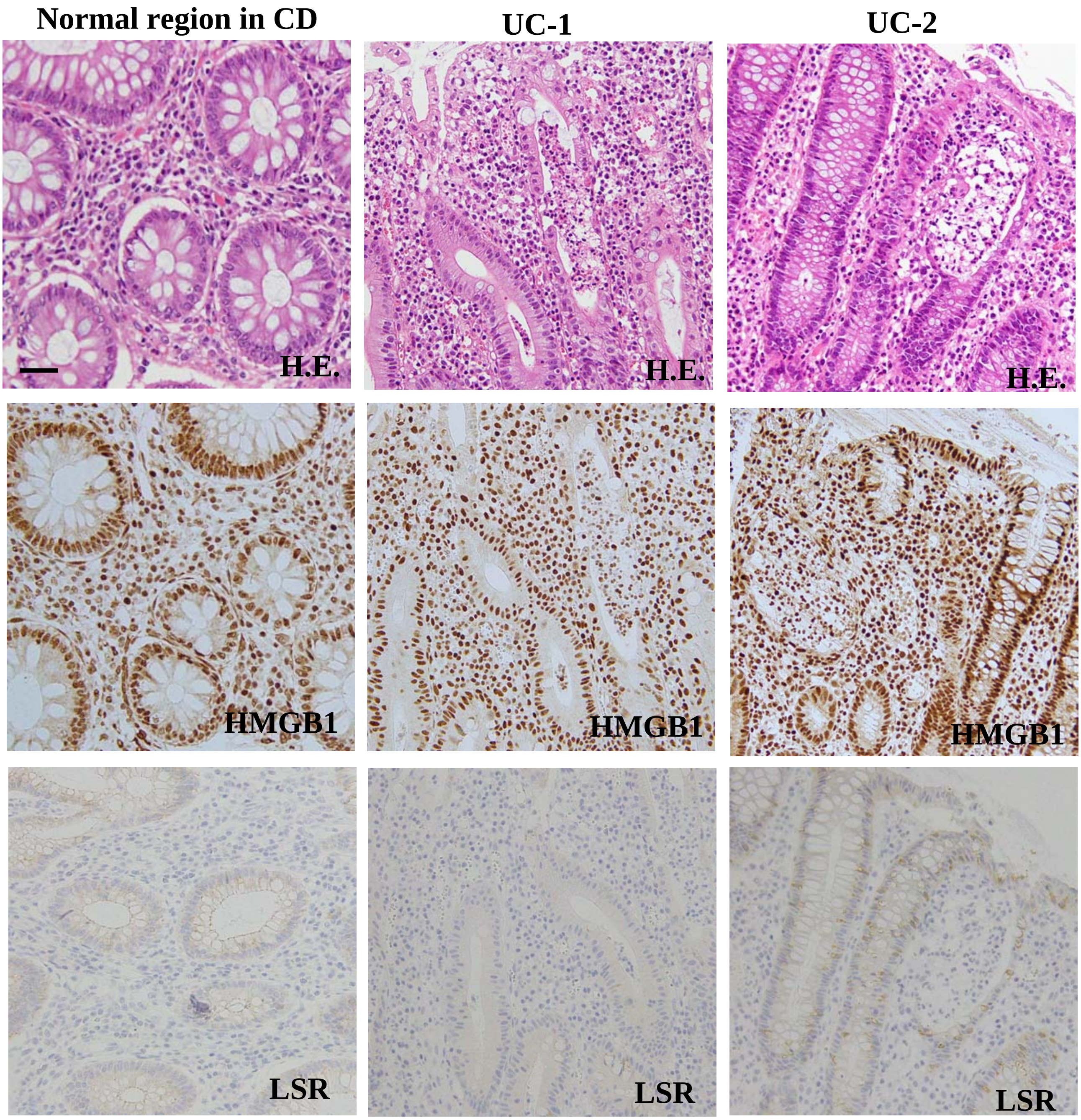

Supplemental Figure 3: Expression and distribution of LSR and HMGB1 in human normal colonic tissues and IBD tissues. Hematoxylin-eosin (H.E.) staining and immunohistochemical staining for HMGB1 and LSR in normal human colonic tissues and colonic those of the IBD colon. CD: Crohn's disease. UC: Ulcerative colitis. Bar: 50 μm.
